# Using your mobile phone for quantitative measurement of spinal motion: a reliability and validity study

**DOI:** 10.1186/1748-7161-10-S1-O29

**Published:** 2015-01-19

**Authors:** Tsz Ping Lam, Bobby Kin Wah Ng, Kwong Man Lee, Lyn Lee Ning Wong, Fiona Wai Ping Yu, Sherry Yin Yu Chan, Man Sang Wong, Jack Chun Yiu Cheng

**Affiliations:** 1 Department of Orthopaedics and Traumatology, The Chinese University of Hong Kong, Hong Kong; 2 Lee Hysan Clinical Research Laboratories, The Chinese University of Hong Kong, Shatin, Hong Kong; 3 Interdisciplinary Division of Biomedical Engineering, The Hong Kong Polytechnic University, Hong Kong; 4 Joint Scoliosis Research Center of the Chinese University of Hong Kong and Nanjing University, China

## Objectives

Measurement of spinal range of movement (ROM) is important for evaluation of patients with spine problems. No convenient method is currently available at routine clinical settings for separate measurement of ROM at the thoracic and lumbar spine. MSKROM is a software application engineered within the Android 4.x.x. OS platform in a mobile phone for measurement of spinal movement using the in-built accelerometer. The objectives of this study were to evaluate the validity and reliability of MSKROM. The validity of the fingertip-to-foor method was also studied and compared with MSKROM.

## Material and methods

Bench validity was checked by measuring the angles from reference angle plate which was a fat surface the inclination of which could be adjusted. Afterwards, 32 healthy female subjects (mean=16.3, SD=1.7 years old) were recruited. They performed spinal movements of (a) full flexion, (b) full extension, (c) full right lateral bending and (d) full left lateral bending. T1, T12, L1 and L5 were registered with the mobile device at various postures and ROM at the thoracic (T1-T12) and lumbar (L1-L5) segment was automatically calculated. Electronic Inclinometer was used as the gold standard. Fingertip-to-floor method was carried out with the distance of finger-tip from the floor measured according to the standard protocol.

## Results

Bench validity of the MSKROM was high (r=1.00, p<0.001). There was excellent concurrent validity of MSKROM when compared against the Electronic Inclinometer with Pearson correlation coefficient ranging from 0.925 to 0.991 except 0.757 at the thoracic extension measurement (all with p<0.001). The intra and inter-rater reliability of MSKROM was also excellent with ICC ranging from 0.873 to 0.997. In contrast, concurrent validity of the fingertip-to-floor method was poor with Pearson correlation coefficient ranging from -0.048 to 0.237 and none of the correlation reached statistical significance.

## Conclusions

The results provided strong evidences the MSKROM installed in a readily available mobile phone with an in-built accelerometer could give valid and reliable measurement of spinal movement in the frontal and sagittal planes. The measurement was simple and fast. Given the inadequacy with the fingertip-to-floor method which is a measure of combined movement at both the hip and the spine, MSKROM can be disseminated for use both at bedside and for clinical researches.

